# 

**Published:** 1885-02-20

**Authors:** 


### TABLE OF CONTENTS.

ORIGINAL AND SELECTED ARTICLES:

Medical and Surgical Uses of Spirits of Turpentine, by J. McF. Gaston, M.D., Professor of Principles and Practice of Surgery in the SOUTHERN MEDICAL COLLEGE, Atlanta Georgia, 41. The Professor and the Quack, by a Georgia Physician, 45. The Property of Alcohol which Allures the Neurotic to Drink, 47. Treatment of Fresh, Open Wounds 50. A New Method for the Treatment of Ingrowing Toe-Nails, 52 On Uterine Hæmorrhage and a New Method of Treatment, 54.

ABSTRACTS AND GLEANINGS:

Climate of Las Vegas, N. Mex; The A. C. E. Mixture, Nitrous Oxide, and Ether, 57. Treatment of Chronic Dysentery by Voluminous Injections of Nitrate of Silver, 58. Muriate of Cocaine in Labor, 59. True Croup; Report of Case of Labor in a Girl aged Eleven Years and Nine Months, 60. Alleged "Vaccine" against Yellow Fever; Gastric Ulcer; Treatment of Nasal Polypi, 61. Treatment of Ringworm; Treatment of Corpulence on Physiological Principles, 62. Lupus; Accidents Produced by Tincture of Arnica, 63. On the Use of Iodoform in Erysipelas, 64 Chronic Laryngeal Catarrh, 65 Hydrobromate of Conia in Epilepsy, 66. On the Therapeutic Uses of Resorcine, 67. Cannibalism; The Oleomargarine War, 68. Indiscriminate Dosing; Treatment of Whooping-Cough; Cannabis Indica—Indian Hemp; Cholera Infantum, 69. Cocaine in Laryngology; Sciatica; Chronic Bronchitis; A Reply to Koch, 70.

SCIENTIFIC ITEMS:

Winds and the Weather; The Oil-Spot, 71. A New Application of the Electric Light; Snow-water Impurities, 72.

PRACTICAL NOTES AND FORMULÆ.

Pleasant Antiperiodic; Tetter Ointment; Compound Hydrastis Ointment, 73. Arnicated Glycerole of Cantharides for the Hair; Angostura Bitters; Compound Syrup of Horehound, 75. Application for Diphtheria; For Nervous Prostration; Rheumatism; Treatment of Boils, 75.

EDITORIALS AND MISCELLANEOUS.

Editorial Notices; The Fordyce Barker Imbroglio, 76. The Lancet; The New Health Bill before Congress; Book Notices, 77. Receipted; Special Notices, 80.

